# Spatiotemporal co-circulation of four dengue serotypes across Mexico, 2020–2025: A space-time scan analysis

**DOI:** 10.1371/journal.pone.0354885

**Published:** 2026-07-31

**Authors:** Oliver Mendoza-Cano, Xóchitl Trujillo, Mónica Ríos-Silva, Jaime Alberto Bricio-Barrios, Agustin Lugo-Radillo, Rosa Tapia-Vargas, Jesús Venegas-Ramírez, Eder Fernando Ríos-Bracamontes, Herguin Benjamin Cuevas-Arellano, Yolitzy Cárdenas, Raúl Aquino-Santos, Iris Anecxi Jiménez-Vieyra, Juan Manuel Uribe-Ramos, Verónica Benites-Godínez, Teresa Evangelina Martínez-Díaz, Mario López-Rojas, Annel García-Solórzano, Efrén Murillo-Zamora

**Affiliations:** 1 Facultad de Ingeniería Civil, Universidad de Colima, Coquimatlán, Mexico; 2 Centro Universitario de Investigaciones Biomédicas, Universidad de Colima, Colima, Mexico; 3 Facultad de Medicina, Universidad de Colima, Colima, Mexico; 4 SECIHTI - Facultad de Medicina y Cirugía, Universidad Autónoma Benito Juárez de Oaxaca, Oaxaca, Mexico; 5 Coordinación de Planeación y Enlace Institucional, Jefatura de Servicios de Prestaciones Médicas, Instituto Mexicano del Seguro Social, Colima, Mexico; 6 Coordinación Auxiliar Médica de Investigación en Salud, Jefatura de Servicios de Prestaciones Médicas, Instituto Mexicano del Seguro Social, Colima, Mexico; 7 Departamento de Medicina Interna, Hospital General de Zona No. 1, Instituto Mexicano del Seguro Social, Villa de Álvarez, Mexico; 8 Facultad de Ciencias, Universidad de Colima, Colima, Mexico; 9 Coordinación General de Investigación, Universidad de Colima, Colima, Mexico; 10 Coordinación de Educación en Salud, Jefatura de Servicios de Prestaciones Médicas, Instituto Mexicano del Seguro Social, Tepic, México; 11 Unidad Académica de Medicina Humana, Universidad Autónoma de Nayarit, Tepic, México; 12 Coordinación Auxiliar Médica de Educación en Salud, Jefatura de Servicios de Prestaciones Médicas, Instituto Mexicano del Seguro Social, Colima, Mexico; 13 Unidad de Investigación en Epidemiología Clínica, Instituto Mexicano del Seguro Social, Villa de Álvarez, Mexico; Universidad San Francisco de Quito, ECUADOR

## Abstract

**Background:**

Mexico has experienced escalating dengue transmission driven by the co‑circulation of four antigenically distinct serotypes (DENV‑1 through DENV‑4). Multi‑serotype transmission is epidemiologically relevant, yet its spatiotemporal patterns at sub‑national resolution remain poorly characterized.

**Methods:**

We analyzed 103,426 PCR‑confirmed, serotyped dengue cases across 1,547 of 2,471 Mexican municipalities from January 2020 to December 2025. Kulldorff’s space‑time scan statistic under a discrete Poisson model was applied independently to each serotype and to all serotypes combined. Co‑circulation was defined as the spatiotemporal overlap of significant clusters from at least two serotypes within the same municipality for ≥ 1 epidemiological week.

**Results:**

We identified 159 statistically significant clusters across all serotypes combined. DENV‑3 was dominant (64.4% of cases; annual incidence 59.9/100,000), consistent with the reemergence of a long‑absent serotype. The 2024–2025 season produced a nationally synchronized epidemic across geographically distant regions. Co‑circulation of at least two serotypes occurred in 1,545 municipalities; 217 experienced simultaneous clustering of all four serotypes. Active co‑circulation was present in 275 of 311 study weeks. Mean pairwise temporal overlap ranged from 8.0 to 12.0 weeks, with maximum overlaps of 29–30 weeks.

**Conclusions:**

Four‑serotype co‑circulation was documented at municipal resolution, concentrated in the Gulf coast, the Yucatán Peninsula, and northeastern Mexico. The 217 municipalities with simultaneous clustering of all four serotypes may represent areas of epidemiological interest for further investigation. This municipality‑level spatiotemporal framework offers operationally relevant resolution for tracking multi‑serotype activity and supporting serotype‑aware dengue monitoring at sub‑national scale.

## Introduction

Dengue virus (DENV) infection has become one of the most important vector-borne public health problems of the 21st century. An estimated 400 million infections occur annually across more than 100 countries [1], and the global burden has grown substantially over the past five decades, driven by urbanization, climate change, and the expanding geographic range of its primary vector, *Aedes aegypti* [2]. The Americas have been particularly affected, accounting for a large and increasing proportion of reported cases worldwide [3].

The existence of four antigenically distinct DENV serotypes (DENV‑1 through DENV‑4) adds epidemiological complexity. Primary infection confers lasting homotypic immunity but only transient cross‑protection against other serotypes [4], allowing multiple serotypes to circulate concurrently. The co‑circulation of serotypes within the same geographic area shapes population‑level transmission patterns and epidemic potential [5]. Understanding where and when serotype co‑circulation occurs is therefore relevant for anticipating shifts in transmission and informing public health planning.

Despite its importance, the spatiotemporal characterization of DENV serotype co-circulation remains limited in many endemic countries [6]. Routine surveillance systems are designed to detect and count cases, not to identify the geographic structure of transmission or the spatiotemporal overlap between serotypes [7]. Aggregate national or state-level reports obscure the fine-grained heterogeneity of dengue dynamics, which operates at the municipal or even neighborhood scale [8].

Space-time scan statistics, originally developed by Kulldorff, offer a rigorous and reproducible framework for identifying clusters of excess incidence in both space and time simultaneously [9], and have been increasingly applied to dengue surveillance data in Asia and Latin America [10,11]. However, their application to serotype-specific and multi-serotype analyses at national scale remains scarce. Alternative spatial approaches, such as Bayesian hierarchical models or spatial autocorrelation metrics, can characterize spatial dependence or estimate smoothed risk surfaces, but they address different inferential goals and are less directly oriented toward detecting discrete spatiotemporal clustering [12,13]. Given our objective of identifying where and when serotype‑specific clustering occurs, the scan statistic provides an appropriate and reproducible analytic framework.

Mexico presents a compelling and underexplored setting for this type of analysis. With over 130 million inhabitants distributed across 2,471 municipalities spanning diverse ecological zones, the country harbors heterogeneous dengue transmission dynamics shaped by geography, climate, and vector ecology. This ecological and demographic diversity produces marked spatial heterogeneity in serotype circulation and epidemic timing, making Mexico an informative setting for examining the geographic structure of multi‑serotype transmission. The General Directorate of Epidemiology (DGE) maintains a national surveillance system with publicly available, municipality‑level records that include laboratory confirmation and serotype information for a substantial proportion of reported cases [14].

The epidemiological period from 2020 to 2025 was characterized by marked serotype turnover and culminated in one of the most severe dengue seasons on record in 2024–2025. A central feature of this period was the regional reemergence of DENV‑3, a serotype whose sustained circulation in the Americas had been largely absent since the late 2000s. The reemergence of DENV‑3 was likely driven by the introduction of a novel Genotype III lineage that had been largely absent from the Americas for more than a decade [15,16], together with a population‑level reduction in pre‑existing DENV‑3 immunity resulting from its prolonged absence in Mexico and the broader region.

The present study aimed to identify and characterize spatiotemporal clusters of dengue incidence in Mexico from 2020 to 2025 using Kulldorff’s space-time scan statistic, applied independently to each of the four DENV serotypes and to all serotypes combined, and to quantify the extent and geographic distribution of serotype co-circulation at the municipal level based on the spatiotemporal overlap of statistically significant serotype-specific clusters. By operating at municipal resolution with serotype-specific resolution, this framework is designed to generate actionable intelligence directly applicable to the prioritization of vector control, clinical surveillance, and vaccine deployment at the sub-national scale.

## Methods

### Study design

We conducted a retrospective observational study to characterize DENV transmission dynamics in Mexico using national epidemiological surveillance records from the DGE. The dataset included the 103,426 polymerase chain reaction (PCR)-confirmed dengue cases with available serotype data and symptom onset between January 1, 2020, and December 13, 2025 (corresponding to epidemiological weeks 1–311 of the study period) as recorded in the publicly available surveillance repository and retrieved on March 18, 2026 [17]. Date of symptom onset served as the primary temporal reference for case ascertainment and analysis. PCR confirmation with serotype assignment was required because the primary analytical objective, serotype-specific and multi-serotype spatiotemporal cluster detection, cannot be addressed with clinically or epidemiologically classified cases lacking laboratory serotype determination.

### Case definition and estimation of population denominators

Confirmed dengue cases were defined as records with a final classification code of 2 (laboratory-confirmed dengue, as specified in the DGE coding scheme for the SINAVE surveillance platform) and a valid serotype entry (1, 2, 3, or 4) in the PCR result field. No age restriction was applied; all age groups were eligible for inclusion.

The raw dataset comprised 1,163,058 records, of which 677,197 (58.2%) were classified as suspected, 235,226 (20.2%) were laboratory-confirmed, and 250,635 (21.6%) were discarded as non-DENV infections. Among laboratory-confirmed cases, 103,426 (44.0%) had serotype data available and constituted the analytical sample. The remaining 131,800 laboratory-confirmed cases (56.0%) lacked serotype assignment; under current DGE guidelines, RT-PCR for serotype identification is performed on a sentinel sample of confirmed cases rather than on all positive specimens, such that the absence of serotype data does not necessarily indicate a failed or indeterminate test but rather reflects the sentinel-based structure of Mexico’s laboratory surveillance network.

Population denominators were derived from the 2020–2025 municipal projections published by the National Population Council (CONAPO) as the sum of sex-specific estimates [18]. Given the six-year study period, the unweighted mean of annual municipal population estimates was used as the denominator for incidence rate calculations, expressed per 100,000 inhabitants.

### Data preparation and geospatial analysis

In the DGE surveillance database, each record contains two separate numeric fields: a two-digit state identifier (CVE_ENT, ranging from 01 to 32) and a three-digit municipality identifier within that state (CVE_MUN, ranging from 001 to 570). These two fields were concatenated to produce a unique five-digit municipal code (e.g., state 06, municipality 002, was the identifier 06002) consistent with the classification scheme of the INEGI, which was used as the common key for all spatial joins and data linkage operations. A vector layer was constructed from the 2025 Geostatistical Framework [19], and municipal centroids were extracted to serve as coordinate references for spatial analysis. All maps presented in were created originally by the research team using the 2025 Geostatistical Framework and municipal shapefiles derived from this public data source, ensuring they contain no third-party copyrighted or proprietary content.

Case data were aggregated by municipality and epidemiological week to produce the case input file (CAS format) required by SaTScan, in which each record specifies the municipality identifier, the epidemiological week, and the corresponding case count. Week 1 (December 29, 2019–January 4, 2020), was defined as the week containing the first confirmed case (January 1, 2020); all subsequent weeks were numbered consecutively. A temporal discontinuity was identified in the weekly sequence: 12 weeks contained no registered cases in any municipality nationwide and therefore did not appear in the dataset. These weeks were retained in the CAS file with zero case counts for all municipalities, ensuring a complete and uninterrupted 311-week temporal sequence for the scan statistic.

All data preparation and geospatial processing steps described above were performed in R version 4.5.2 (R Foundation for Statistical Computing, Vienna, Austria).

### Spatiotemporal statistical analysis

Spatiotemporal clusters of dengue incidence were identified using Kulldorff’s space-time scan statistic, implemented in SaTScan version 10.3.3 (Martin Kulldorff and Information Management Services Inc., Boston, MA, USA). In this analysis, a cluster was defined as a cylindrical space-time window, comprising a circular geographic base and a temporal interval, containing a significantly higher number of observed dengue cases than expected under the null hypothesis of spatial and temporal randomness, as evaluated by the log-likelihood ratio (LLR). A discrete Poisson model was specified, under which the expected number of cases in each municipality is proportional to its population at risk.

The scanning window was defined as a cylinder comprising a circular geographic base and a temporal dimension. Maximum spatial and temporal window sizes were set to 10% of the population at risk and 10% of the study period, respectively. This conservative parameterization was chosen to favor the detection of geographically and temporally circumscribed clusters, consistent with the focal transmission patterns expected for dengue in Mexico, and to avoid the identification of implausibly large clusters with limited epidemiological interpretability. This approach has been previously shown to improve cluster detection sensitivity in spatiotemporal analyses of dengue compared to the default 50% threshold [20]. Time aggregation was set to one unit corresponding to one epidemiological week, with the study period expressed as weeks 1–311. A minimum of two cases was required for cluster reporting.

All analyses were retrospective; purely spatial and purely temporal analyses were not performed. Statistical significance was evaluated using 999 Monte Carlo replications, and results were reported under a no geographical overlap criterion. P-values were computed using the Default Combination method in SaTScan: when the observed LLR exceeded the maximum LLR across all 999 replications, significance was assessed analytically from the asymptotic chi-squared distribution of the LLR statistic rather than as a Monte Carlo proportion; otherwise, the Monte Carlo-derived p-value was reported. This procedure simultaneously adjusts for scanning over all possible spatial and temporal window configurations, inherently accounting for multiple testing without requiring post-hoc correction.

For each detected cluster, the following metrics were reported: the observed-to-expected case ratio (O/E), the relative risk (RR), defined as the ratio of the incidence rate within the cluster to that outside it, and the log-likelihood ratio (LLR), which serves as the test statistic for evaluating cluster significance under the Poisson model.

Data management and geospatial processing were performed using the R packages tidyverse, sf, lubridate, and data.table. To ensure full reproducibility, the SaTScan parameter file and all input files (case [CAS], population [POP], and geographic coordinates [GEO]) are publicly available as open data. Five sets of CAS files were constructed: one aggregating all serotypes combined, and one for each DENV serotype individually (DENV-1 through DENV-4).

A post-hoc verification was performed to confirm that no significant cluster had its start or end week coinciding with any of the 12 epidemiological weeks with zero case registrations.

### Co-circulation analysis of DENV serotypes

Co-circulation was defined as the spatiotemporal co-occurrence of statistically significant clusters (p < 0.05) from at least two distinct DENV serotypes within the same municipality, with a minimum temporal overlap of one complete epidemiological week (≥ 7 days). This threshold was selected because the epidemiological week is the minimum temporal unit of aggregation in the case file; overlaps of shorter duration fall below the resolution of the surveillance data and cannot be interpreted as evidence of genuine simultaneous transmission. This analysis was conducted by integrating the outputs of four independent serotype-specific SaTScan analyses (DENV-1 through DENV-4), rather than a single joint analysis. For each municipality, all pairwise combinations of serotype-specific clusters were evaluated. A pair of clusters belonging to serotypes i ≠ j was classified as temporally overlapping if the following condition was satisfied:


$$\begin{array}{c} min(t\_(end,i),\;t\_(end,j)\;)-max(t\_(start,i),t\_(start,j)\;)+\text{1}\;\geq \text{1} \end{array}$$

where $t_{start}$ and $t_{end}$ denote the first and last epidemiological week of each cluster, respectively. The total number of co-circulating serotypes per municipality was subsequently computed as the count of distinct serotypes involved in at least one such overlapping pair.

Municipalities were subsequently classified into five mutually exclusive categories: (i) no reported cases, defined as municipalities absent from the dengue case registry with no detected cluster for any serotype; (ii) cases without co-circulation, defined as municipalities with at least one reported case but no statistically significant spatiotemporal cluster overlap between any two serotypes, encompassing municipalities with cases attributable to a single serotype, those with multi-serotype cases whose clusters did not meet the temporal overlap criterion, and those with confirmed cases in which no cluster reached statistical significance for any serotype; (iii) co-circulation of two serotypes; (iv) co-circulation of three serotypes; and (v) co-circulation of four serotypes. Categories 3–5 reflect increasing levels of simultaneous serotype activity. Municipality codes were standardized across datasets using the five-digit coding scheme described above.

### Ethical considerations

Given the anonymous and open-access nature of the data, institutional review board oversight and informed consent were not required.

## Results

**Fig 1:**
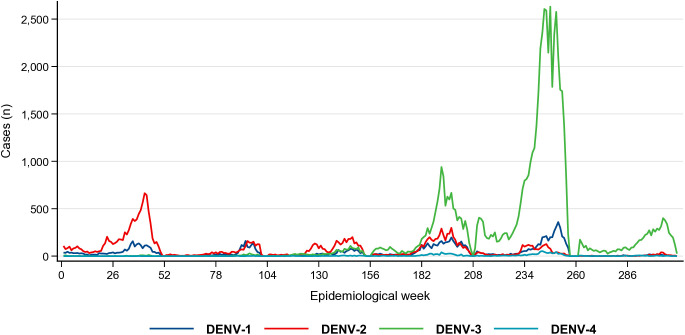
Weekly distribution of dengue virus cases by serotype (DENV-1 through DENV-4) in Mexico, 2020–2025. Each row represents one dengue virus serotype (DENV-1 through DENV-4). Color intensity reflects the weekly case count on a log_10_ (n + 1) scale.

The 103,426 PCR-confirmed dengue cases were distributed across 1,547 of 2,471 Mexican municipalities over the 311-week study period, yielding a mean annual incidence rate of 93.0 cases per 100,000 population based on a national population denominator of 130,619,344. The temporal distribution of cases by serotype is presented in Fig 1. DENV-3 was responsible for most cases (n = 66,589; 64.4%), followed by DENV-2 (n = 22,301; 21.6%), DENV-1 (n = 13,182; 12.7%), and DENV-4 (n = 1,354; 1.3%).

### Space-time cluster analysis: All serotypes combined

The retrospective space-time scan analysis identified 159 statistically significant clusters (p < 0.05) across all four serotypes, out of 192 detected: one most likely cluster and 158 secondary clusters. Of these, 80 clusters reached p < 1 × 10 ^−^ ¹⁷, spanning multiple geographic regions and epidemic periods across the country. Clusters were ranked by log-likelihood ratio (LLR) in descending order, with Cluster 1 representing the most likely cluster. It was centered at 20.85°N, 104.83°W with a radius of 286.33 km, covering municipalities predominantly located in western Mexico (Jalisco, Nayarit, Colima, and Michoacán), with extensions into central Mexico (Zacatecas and Aguascalientes). This cluster was active during weeks 237–255 (October 13, 2024–February 22, 2025) and contained 13,241 observed cases against only 629.68 expected (O/E ratio 21.03; RR 23.97; LLR 28,526.3). It was the single largest dengue event detected in the entire dataset.

The first secondary cluster (Cluster 2) was active during weeks 229–254 (August 18, 2024–February 9, 2025) and encompassed municipalities along the Pacific coast and highlands of southern Mexico (Guerrero and Oaxaca), with extensions into south-central Mexico (Puebla, Morelos, and the State of México; 9,100 cases, O/E: 14.35; RR: 15.64; LLR: 16,134.1). A third large cluster (Cluster 3) spanned northern Mexico (Coahuila, Chihuahua, Nuevo León, Tamaulipas, and Durango) and north-central Mexico (Zacatecas) during weeks 240–255 (November 3, 2024–February 16, 2025) (7,410 cases; O/E 16.47; RR 17.66; LLR 14,039.1).

Cluster 4 was confined to southeastern Mexico (Quintana Roo, Yucatán, and Campeche) and temporally distinct from other clusters, active during weeks 180–204 (October 20, 2022–April 13, 2023), with 6,212 cases recorded in a population of 4,884,395 (O/E 19.98; RR: 21.19; LLR 12,874.7). Cluster 5 covered the same southern Pacific axis (Guerrero and Oaxaca) with extensions into south-central Mexico (Puebla), but during a non-overlapping time window (weeks 183–213; November 10, 2022–July 27, 2023), accounting for 5,405 cases (O/E 7.77; RR 8.14; LLR 6,482.5); the recurrence of significant clustering in this region across distinct periods suggests persistent spatio-temporal transmission foci. Cluster 6 spanned west-central Mexico (Guanajuato and Querétaro) and western Mexico (Michoacán) during weeks 233–255 (September 15, 2024–February 16, 2025), recording 3,607 cases (O/E 9.65; RR 9.96; LLR 4,994.4).

**Fig 2:**
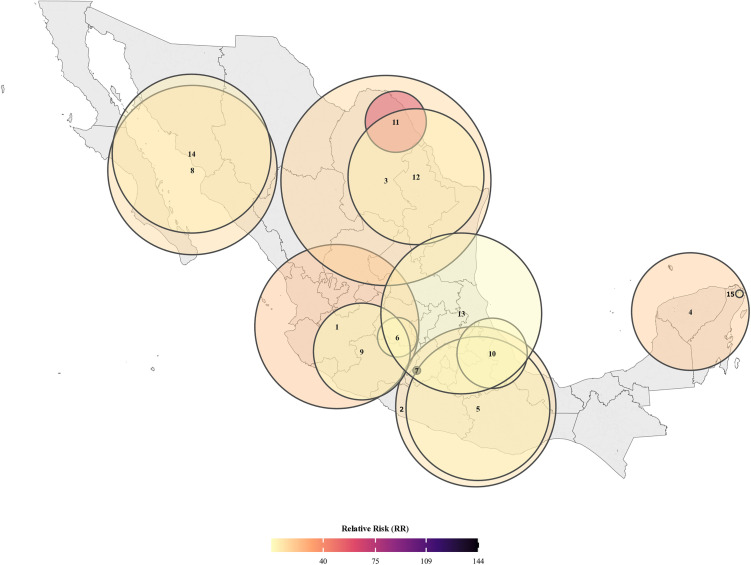
Fifteen most likely spatiotemporal clusters of dengue incidence across all serotypes combined, Mexico, 2020–2025. Each circle represents a statistically significant space-time cluster (p < 0.05, evaluated against 999 Monte Carlo replications) detected by Kulldorff’s scan statistic under a discrete Poisson model. Circle area is proportional to the geographic radius of the cluster. Fill color reflects the relative risk (RR) of dengue incidence within the cluster compared to the surrounding area, as indicated by the color scale. Numbers identify cluster rank by log-likelihood ratio (LLR), with Cluster 1 representing the most likely cluster. Population denominators are derived from CONAPO 2020–2025 municipal projections.

An intense but geographically compact cluster (Cluster 7) was identified within two municipalities of south-central Mexico (State of México), active during weeks 123–145 (April 8–September 11, 2021). Despite a small population at risk (121,598), it registered 1,016 cases against an expectation of 7.12 (O/E 142.68; RR 144.09; LLR 4,036.1), indicating a concentrated local epidemic. The remaining significant clusters (8–159) spanned states across northwestern Mexico (Baja California Sur, Sonora, and Sinaloa), southeastern Mexico (Veracruz, Chiapas, and Tabasco), northeastern Mexico (Tamaulipas, San Luis Potosí, and Hidalgo), and the Caribbean coast of southeastern Mexico (Quintana Roo), collectively confirming the multifocal and heterogeneous nature of dengue transmission across the country during the study period. The 15 most likely clusters are presented in Fig 2.

Post-hoc verification confirmed that none of the 159 significant clusters was temporally truncated by surveillance gaps.

### Serotype-specific space-time cluster analysis: DENV-1

**Fig 3:**
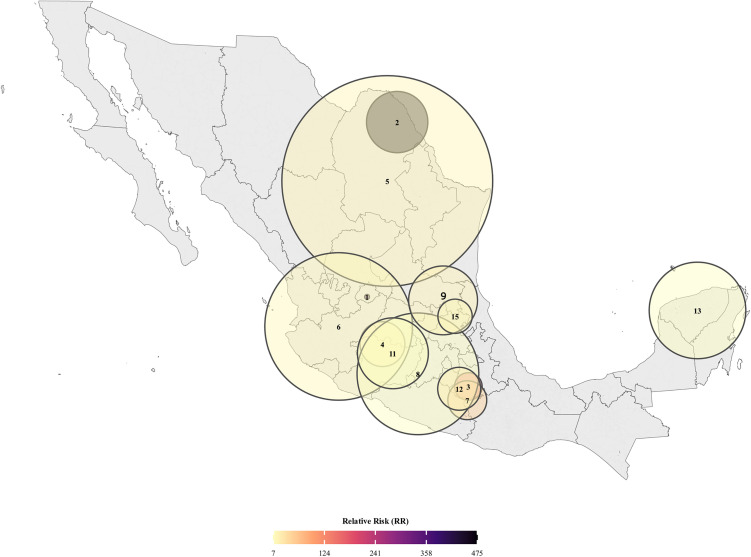
Fifteen most likely spatiotemporal clusters of dengue incidence for DENV-1, Mexico, 2020–2025. Each circle represents a statistically significant space-time cluster (p < 0.05, evaluated against 999 Monte Carlo replications) detected by Kulldorff’s scan statistic under a discrete Poisson model. Circle area is proportional to the geographic radius of the cluster. Fill color reflects the relative risk (RR) of dengue incidence within the cluster compared to the surrounding area, as indicated by the color scale. Numbers identify cluster rank by log-likelihood ratio (LLR), with Cluster 1 representing the most likely cluster. Population denominators are derived from CONAPO 2020–2025 municipal projections.

The serotype-specific analysis for DENV-1 identified a total of 87 clusters, of which 15 most likely are presented in Fig 3. DENV-1 contributed 13,182 cases (annual incidence 11.9/100,000) over the 311-week study period. The most likely cluster (Cluster 1) was a compact, high-intensity focus comprising only two municipalities in central Mexico (Aguascalientes), active during epidemiological weeks 243–256 (November 24, 2024–February 23, 2025). Despite its small geographic footprint (radius 20.46 km), it accumulated 1,558 cases against an expectation of 5.16 (O/E 302.03; RR 342.38; LLR 7,439.5; p < 1 × 10 ^−^ ¹⁷), reflecting a highly localized outbreak. Cluster 2 identified a focused outbreak in northern Mexico (Coahuila) during weeks 89–99 (August 6–October 15, 2020), recording 791 cases (O/E 446.74; RR 475.20; LLR 4,061.6). The characteristics of the remaining significant clusters are provided in Supplementary Dataset.

### Serotype-specific space-time cluster analysis: DENV-2

**Fig 4:**
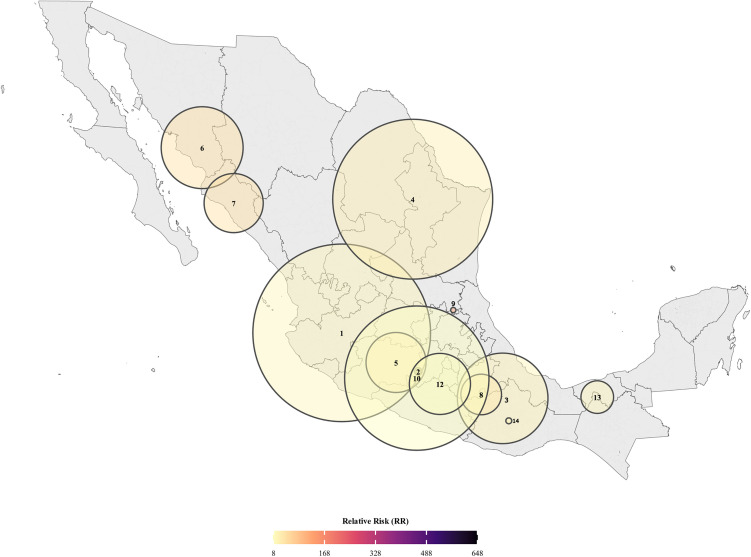
Fifteen most likely spatiotemporal clusters of dengue incidence for DENV-2, Mexico, 2020–2025. Each circle represents a statistically significant space-time cluster (p < 0.05, evaluated against 999 Monte Carlo replications) detected by Kulldorff’s scan statistic under a discrete Poisson model. Circle area is proportional to the geographic radius of the cluster. Fill color reflects the relative risk (RR) of dengue incidence within the cluster compared to the surrounding area, as indicated by the color scale. Numbers identify cluster rank by log-likelihood ratio (LLR), with Cluster 1 representing the most likely cluster. Population denominators are derived from CONAPO 2020–2025 municipal projections.

DENV-2 was the second most prevalent serotype (22,301 total cases; annual rate 20.1/100,000). The 15 most likely space-time clusters are presented in Fig 4. The most likely cluster (Cluster 1) was a large, multi-state focus centered at 19.98°N, 105.16°W spanning western Mexico (Jalisco, Colima, Nayarit, Michoacán, and Sinaloa) and central Mexico (Zacatecas and Aguascalientes), active during weeks 21–49 (May 19–November 28, 2020), recording 3,489 cases against 206.94 expected (O/E 16.86; RR 19.80; LLR 6,831.0; p < 1 × 10 ^−^ ¹⁷). Cluster 2 was a compact but intense focus restricted to four municipalities in south-central Mexico (State of México; radius 21.65 km), active during weeks 121–145 (March 25–September 11, 2021), recording 1,031 cases against 1.67 expected (O/E 617.78; RR 647.68; LLR 5,620.1), representing the highest relative risk of any cluster across all serotype-specific analyses. The characteristics of the remaining significant clusters are summarized in Supplementary Dataset.

### Serotype-specific space-time cluster analysis: DENV-3

**Fig 5:**
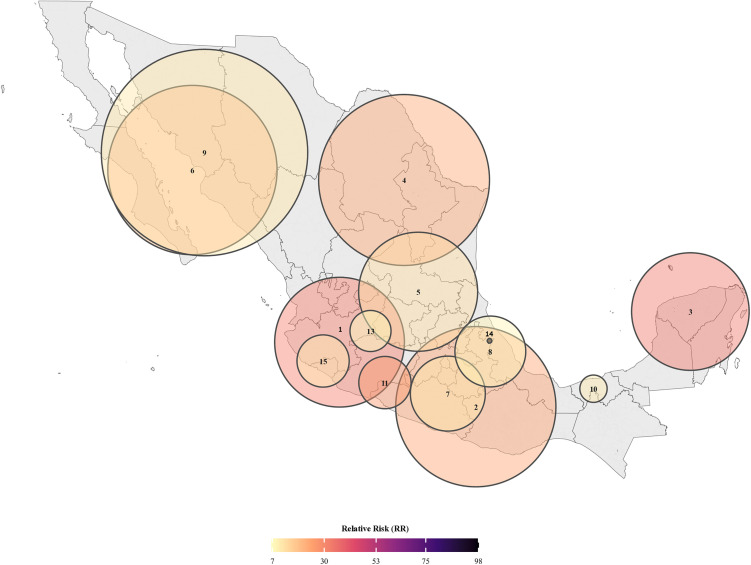
Fifteen most likely spatiotemporal clusters of dengue incidence for DENV-3, Mexico, 2020–2025. Each circle represents a statistically significant space-time cluster (p < 0.05, evaluated against 999 Monte Carlo replications) detected by Kulldorff’s scan statistic under a discrete Poisson model. Circle area is proportional to the geographic radius of the cluster. Fill color reflects the relative risk (RR) of dengue incidence within the cluster compared to the surrounding area, as indicated by the color scale. Numbers identify cluster rank by log-likelihood ratio (LLR), with Cluster 1 representing the most likely cluster. Population denominators are derived from CONAPO 2020–2025 municipal projections.

DENV-3 was the dominant serotype throughout the study period, accounting for 66,589 cases (annual incidence 59.9/100,000). The 15 most likely space-time clusters are presented in Fig 5. The most likely cluster (Cluster 1) was centered at 18.89°N, 105.03°W, with a radius of 334.23 km, covering municipalities across western Mexico (Jalisco, Colima, Nayarit, and Michoacán) and central Mexico (Zacatecas), active during weeks 232–255 (September 8, 2024–February 16, 2025). It recorded 12,233 cases against 466.82 expected (O/E 26.20; RR 31.88; LLR 29,301.2; p < 1 × 10 ^−^ ¹⁷), the highest LLR of any cluster across all serotype-specific analyses, underscoring the dominance of DENV-3 during the final phase of the study period. The temporal overlap between Clusters 1, 2, and 3 (weeks 229–255; August 18, 2024–February 16, 2025) is consistent with a synchronous multi-regional DENV-3 epidemic unfolding simultaneously across Mexico’s Pacific coast, central highlands, and Caribbean coast. The characteristics of the remaining significant clusters are provided in Supplementary Dataset.

### Serotype-specific space-time cluster analysis: DENV-4

**Fig 6:**
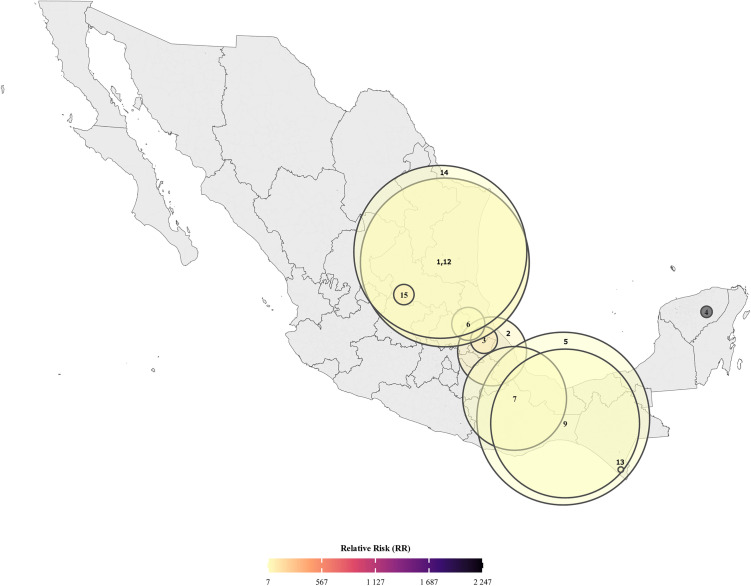
Fifteen most likely spatiotemporal clusters of dengue incidence for DENV-4, Mexico, 2020–2025. Each circle represents a statistically significant space-time cluster (p < 0.05, evaluated against 999 Monte Carlo replications) detected by Kulldorff’s scan statistic under a discrete Poisson model. Circle area is proportional to the geographic radius of the cluster. Fill color reflects the relative risk (RR) of dengue incidence within the cluster compared to the surrounding area, as indicated by the color scale. Numbers identify cluster rank by log-likelihood ratio (LLR), with Cluster 1 representing the most likely cluster. Population denominators are derived from CONAPO 2020–2025 municipal projections.

DENV-4 was the least prevalent serotype during the study period, with 1,354 total cases and an annual incidence of 1.2 per 100,000 population. Despite the low absolute case count, the space-time scan analysis identified spatially and temporally well-defined clusters with high relative risks, reflecting concentrated localized outbreaks (Fig 6). The most likely cluster (Cluster 1) was centered at 23.78°N, 98.04°W with a radius of 325.57 km, encompassing municipalities across northeastern Mexico (Tamaulipas and Nuevo León), north-central Mexico (San Luis Potosí), east-central Mexico (Hidalgo and Querétaro), and the Gulf coast (Veracruz), active during weeks 236–254 (October 6, 2024–February 2, 2025), recording 356 cases against 7.05 expected (O/E 50.48; RR 68.14; LLR 1,096.9; p < 1 × 10 ^−^ ¹⁷). Cluster 12 shared an identical centroid and nearly identical geographic extent with Cluster 1, but was active during an earlier, non-overlapping window (weeks 228–234; August 11–September 22, 2024), recording 23 cases (O/E 9.30; RR 9.44; LLR 30.9; p < 0.001), consistent with progressive amplification of DENV-4 transmission preceding the main outbreak. The characteristics of the remaining significant clusters are provided in Supplementary Dataset.

### Multi-serotype transmission: Municipality-level patterns

**Fig 7:**
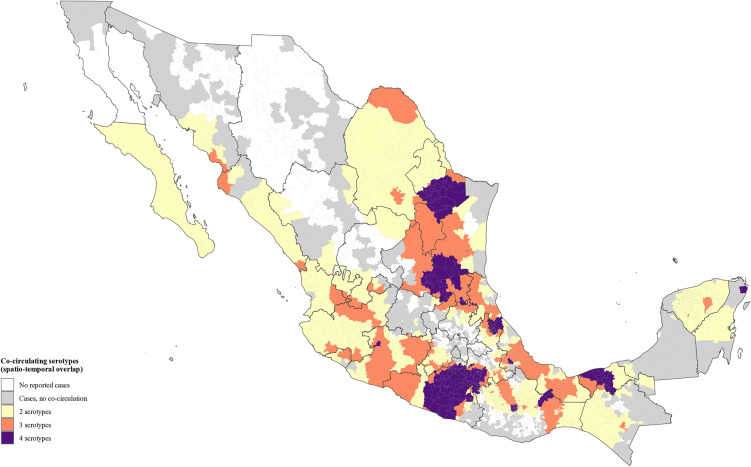
Municipal-level spatiotemporal co-circulation of dengue virus serotypes, Mexico, 2020–2025. Municipalities were classified into five mutually exclusive categories according to the number of DENV serotypes with statistically significant spatiotemporal clusters (p < 0.05, evaluated against 999 Monte Carlo replications) from four independent serotype-specific SaTScan analyses (DENV-1 through DENV-4), out of 2,471 total Mexican municipalities. Co-circulation was defined as the simultaneous presence of significant clusters from at least two distinct serotypes within the same municipality, with a minimum temporal overlap of one complete epidemiological week (≥ 7 days).

Co-circulation was active across 275 of the 311 study weeks, with peak activity recorded at week 197 (March 2, 2023), during which 2,869 serotype pairs were simultaneously active. Weeks 241–246 (November 3–December 8, 2024) concentrated six of the ten highest-activity weeks observed across the entire study period, indicating a sustained episode of intense multi-serotype transmission during the final phase of the study (Fig 7).

At the municipal level, co-circulation patterns varied in the number of serotypes involved (Fig 7; Table 1). Co-circulation of exactly two serotypes was the most prevalent pattern (n = 871 municipalities), with DENV-2/DENV-4 (277 municipalities) and DENV-1/DENV-2 (258 municipalities) as the most common exclusive pairings. Three-serotype co-circulation was identified in 457 municipalities, predominantly involving DENV-2/DENV-3/DENV-4 (162 municipalities), DENV-1/DENV-2/DENV-3 (135 municipalities), and DENV-1/DENV-3/DENV-4 (132 municipalities). Concurrent clustering of all four serotypes was documented in 217 municipalities, concentrated in the Gulf coast (Veracruz, Puebla, and Morelos), southeastern Mexico (Chiapas, Quintana Roo, Yucatán, and Campeche), and northeastern Mexico (Tamaulipas and Nuevo León). The complete distribution of exclusive serotype combinations and pairwise overlap metrics is presented in Table 1 and 2, respectively.

**Table 1:** Serotype co-circulation combinations by municipality, Mexico, 2020–2025.

Serotype combination	Municipalities (n)
***Two serotypes***	
DENV-2 + DENV-4	277
DENV-1 + DENV-2	258
DENV-1 + DENV-3	196
DENV-1 + DENV-4	62
DENV-3 + DENV-4	48
DENV-2 + DENV-3	30
***Three serotypes***	
DENV-2 + DENV-3 + DENV-4	162
DENV-1 + DENV-2 + DENV-3	135
DENV-1 + DENV-3 + DENV-4	132
DENV-1 + DENV-2 + DENV-4	28
***Four serotypes***	
DENV-1 + DENV-2 + DENV-3 + DENV-4	217

Abbreviations: DENV, dengue virus.

Co-circulation was defined as the spatiotemporal co-occurrence of statistically significant clusters (p < 0.05) from at least two distinct DENV serotypes within the same municipality, with a minimum temporal overlap of one complete epidemiological week (≥ 7 days). Categories are mutually exclusive; municipalities are counted in one row only.

**Table 2:** Pairwise serotype cluster overlap by municipality, Mexico, 2020–2025.

Serotype pair	Municipalities (n)	Mean overlap (weeks)	Maximum overlap (weeks)
DENV-1 + DENV-2	631	10.2	30
DENV-2 + DENV-4	568	11.7	26
DENV-3 + DENV-4	528	9.7	26
DENV-1 + DENV-3	526	9.4	29
DENV-1 + DENV-4	385	12.0	29
DENV-2 + DENV-3	259	8.0	26

Abbreviations: DENV, dengue virus.

Co-circulation was defined as the spatiotemporal co-occurrence of statistically significant clusters (p < 0.05) from at least two distinct DENV serotypes within the same municipality, with a minimum temporal overlap of one complete epidemiological week (≥ 7 days). Pairwise overlap counts are not mutually exclusive: municipalities where more than two serotypes co-circulated are counted in multiple rows.

## Discussion

This study provides a national-scale, serotype-resolved spatiotemporal characterization of dengue transmission in Mexico using Kulldorff’s space-time scan statistic applied independently to each of the four DENV serotypes over a six-year period spanning two major epidemic cycles. The analysis identified 159 statistically significant clusters across all serotypes combined, documented the spatiotemporal footprint of the DENV-3 reemergence that defined the 2024–2025 epidemic season, and quantified the geographic extent of serotype co-circulation at the municipal level.

The most striking finding is the synchronous activation of large, high-intensity clusters across geographically distant regions during weeks 229–255 (August 2024–February 2025), involving three of the four serotypes simultaneously. The most likely cluster overall and the highest-LLR DENV-3 cluster were both centered in the western Pacific axis (Jalisco, Colima, Nayarit, Michoacán), temporally coincident with major clusters in the southern Pacific corridor (Guerrero, Oaxaca) and northern Mexico (Coahuila, Chihuahua, Nuevo León, Tamaulipas), pointing to a nationally synchronized epidemic in which rapid DENV-3 spread amplified transmission already sustained by endemic DENV-1 and DENV-2 circulation. This simultaneity across climatically and ecologically distinct regions suggests a population-level condition created by DENV-3’s prolonged absence from Mexico and the broader Americas, a serotype that can go undetected for over a decade, leaving populations with limited recent exposure [21].

Phylogeographic evidence suggests the GIII-American-II lineage was introduced from the Indian subcontinent around 2019 and circulated cryptically for roughly three years before detection in 2022 [15]; by 2023 DENV-3 had become predominant in a largely naïve population, prompting a 2024 PAHO epidemiological alert for its regional resurgence [22]. The magnitude of the clusters detected (Cluster 1 for DENV-3: O/E 26.20, LLR 29,301.2, the highest of any cluster across all serotype-specific analyses) is quantitatively consistent with rapid transmission into a population with limited circulating immunity.

DENV-3’s dominance (64.4% of serotyped cases; annual incidence 59.9/100,000) was reflected in both case volume and geographic breadth: unlike DENV-1 and DENV-2, whose clusters concentrated in specific regional foci, DENV-3 clusters spanned every major geographic zone, including historically low-incidence areas of northern Mexico, consistent with the broader pattern that extended absences from circulation are followed by larger, more geographically widespread outbreaks [5,23]. This temporal staggering also explains the apparent predominance of DENV-2/DENV-4 and DENV-1/DENV-2 pairings among two-serotype co-circulation municipalities (Table 1) despite DENV-3’s overall dominance: DENV-1, DENV-2, and DENV-4 circulated endemically during 2020–2022, allowing their geographic overlap to accumulate over multiple years, whereas DENV-3 co-circulation, though geographically expansive, emerged as a more recent and temporally concentrated phenomenon in 2023–2025.

Despite the national synchrony of the 2024–2025 epidemic, dengue transmission showed marked spatial heterogeneity at the sub-national scale, with several geographic foci recurring across serotypes and epidemic periods, suggesting structural transmission determinants that transcend any single serotype or cycle. The southern Pacific corridor (Guerrero, Oaxaca, Puebla) generated significant clusters for DENV-1, DENV-2, and DENV-3 across non-overlapping windows; the Yucatán Peninsula produced intense, geographically confined clusters for DENV-3 (O/E 30.30) and DENV-4 (O/E 2,207.51), consistent with its historically high endemicity and sustained *Aedes aegypti* presence; and the State of México yielded high O/E ratios for DENV-1, DENV-2, and DENV-3 within compact foci, a pattern warranting attention given its metropolitan context. This recurrence points to determinants operating independently of the circulating serotype, likely reflecting built-environment characteristics that shape vector populations (housing quality, drainage, heat islands) together with human movement patterns that drive viral dispersal [24]; fine-scale urban population structure strongly determines dengue’s temporal patterns at coarser resolutions [8], and urbanization is robustly associated with vector production, density, and transmission [25]. Because national-level reports obscure this geographic concentration, space-time scan analysis offers actionable intelligence for prioritizing vector control and surveillance, indicating that a disproportionate share of the national burden concentrates in a relatively small number of repeatedly high-risk municipalities.

A clear illustration of mobility-driven transmission is the appearance of major tourist destinations among the municipalities with four-serotype co-circulation in Fig 7, including Benito Juárez (Cancún) and Tulum in Quintana Roo, Oaxaca de Juárez, and the area surrounding Chichén Itzá in Yucatán. Tourism and travel are recognized mechanisms for introducing and mixing dengue serotypes in Mexico [26], with viremic travelers repeatedly seeding distinct serotypes into receptive areas with competent vectors; the concentration of co-circulation in these destinations is consistent with their role as mobility hubs, though surveillance data alone cannot establish directionality without genomic confirmation [27].

DENV-4 accounted for only 1.3% of all serotyped cases, yet the space-time scan analysis identified clusters with relative risks markedly higher than those observed for more prevalent serotypes. The most extreme example was a 24-case cluster in the Yucatán Peninsula during weeks 184–193 (late 2022 to early 2023) with an O/E ratio exceeding 2,200 and an RR of 2,247 from a population of only 32,618. This illustrates both the epidemiological pattern typical of rare serotypes circulating in highly localized, geographically circumscribed settings, and a key analytical advantage of the space-time scan approach over standard incidence mapping: by conditioning case counts on the local population at risk and evaluating statistical significance through likelihood ratio testing, the method identifies epidemiologically meaningful foci that would be numerically invisible in national incidence reports. The scan statistic’s distinctive feature is its ability to detect the geographic location of clusters while providing statistical significance, a capability that purely descriptive mapping techniques do not offer [27].

The biphasic temporal distribution of DENV-4 clusters, with distinct waves in 2022–2023 and 2024–2025, is consistent with this serotype’s known epidemiological history: DENV-4 caused epidemics in Nicaragua in the early 1990s before becoming virtually unreported for decades, reemerging in 2022 to co-dominate epidemics alongside other serotypes as part of a broader pattern of prolonged absence followed by abrupt reappearance across Central America [28]. This emergence coincided regionally with the reemergence of DENV-3, reflecting a wider shift in serotype circulation dynamics across the Americas [29]. Similarly, the temporal gap between DENV-3 clustering in the Yucatán Peninsula (weeks 180–204, 2022–2023) and the Pacific coast/central highlands (weeks 232–255, 2024–2025) is consistent with either sequential geographic spread or independent introduction events at different time points, a pattern documented elsewhere for dengue serotypes and indistinguishable from directional spread based on cluster timing alone [30]. Whether these represent successive introductions linked to regional serotype realignment or distinct spread events cannot be determined from epidemiological surveillance alone; phylogeographic analysis of DENV-3 and DENV-4 sequences from the implicated regions would be needed to reconstruct these transmission pathways and represents a priority for future genomic studies [31].

A central objective of this study was to characterize serotype co-circulation, the spatiotemporal overlap of distinct serotype-specific clusters within the same municipality, as a proxy indicator of the population-level opportunity for sequential heterotypic infection. The finding that 275 of 311 study weeks exhibited active co-circulation, and that 217 municipalities experienced simultaneous significant clustering of all four serotypes, indicates that these ecological conditions were documented at municipal resolution across a substantial fraction of the country [32,33]. Four-serotype co-circulation was geographically concentrated in three sub-national regions: the Gulf coast (Veracruz, Puebla, Morelos), the Yucatán Peninsula (Chiapas, Quintana Roo, Yucatán, Campeche), and northeastern Mexico (Tamaulipas, Nuevo León), where this pattern was most intense during the study period.

Because this study operates at the ecological level, co-circulation events cannot be directly linked to individual-level immunological outcomes or clinical severity, and the following interpretation is therefore framed at the population level. The duration of co-circulation is independently relevant to this inference: mean pairwise temporal overlap ranged from 8.0 weeks (DENV-2/DENV-3) to 12.0 weeks (DENV-1/DENV-4), with maximum overlaps of 29–30 weeks for pairs involving DENV-1. Windows of simultaneous circulation of this magnitude increase the population-level probability of sequential heterotypic exposure within the same municipality [34,35]. The spatiotemporal framework developed here provides the geographic and temporal coordinates needed to design studies linking co-circulation patterns to clinical outcomes using existing surveillance records, bridging the ecological level of this analysis to individual-level health outcomes.

Beyond characterizing the geography of transmission, this serotype-resolved spatiotemporal framework carries direct operational value. Because vector-control resources are finite, localizing serotype-specific foci at municipal resolution allows interventions to be directed toward areas of recurrent elevated risk rather than distributed uniformly across administrative units; surveillance coupling case detection with serotype identification has been shown to refine the spatial targeting of vector control during active epidemics [36]. Documenting which serotypes circulate, and where they co-occur, also informs the anticipation of transmission shifts, since continuous serotype monitoring is increasingly recognized as essential in hyperendemic settings [29]. This is particularly relevant as tetravalent dengue vaccines move toward broader deployment, given that regional serotype circulation patterns and the local dominance of specific serotypes, such as the DENV-3 predominance documented here, bear on the anticipated population-level benefit and geographic prioritization of vaccination programs [37]. Future prospective studies linking the multi-serotype co-circulation patterns documented here to individual-level clinical outcomes, serological status, and disease severity would provide empirical evidence for the relationship between heterotypic dengue exposure and severe dengue manifestations [38]; the municipalities identified as sites of sustained four-serotype co-circulation represent natural laboratory settings for such investigation.

These operational and translational implications rest on several methodological strengths of the present study: a national, publicly available, municipality-level dataset with six years of follow-up and individual-level serotype information, unusual for the dengue literature in Latin America; complete reproducibility, with SaTScan parameter files and all input datasets released as open data; a co-circulation framework operationalized through a precise temporal overlap criterion that avoids the ambiguity of qualitative assessments based on aggregate case counts; and a conservative 10% maximum window parameterization, supported by prior methodological evidence in dengue-specific contexts [39], which favors circumscribed, epidemiologically interpretable foci over implausibly large geographic aggregations.

These strengths should nonetheless be considered alongside several limitations. The restriction of the analytical sample to PCR-confirmed, serotyped cases (103,426 of 235,226 laboratory-confirmed cases; 44.0%) is an important consideration. Serotyping coverage varied across states (range 14.0%–84.4%), though the correlation between state-level case volume and serotyping rate was negligible (r = −0.16), suggesting that geographic bias in serotype ascertainment was not systematically driven by dengue burden. Only two states showed coverage below 20% (Chiapas, 14.0%; Hidalgo, 14.6%); although both still contributed statistically significant clusters, their low serotyping coverage means that the true case burden and serotype distribution in these areas may be substantially under-ascertained, and clusters detected in low-coverage regions should be interpreted with corresponding caution. Municipalities with limited laboratory infrastructure remain underrepresented in the analytical sample, and the geographic distribution of detected clusters should therefore be interpreted as a picture of where serotype-confirmed dengue was concentrated, not necessarily of where total dengue burden was highest.

Our use of independent serotype‑specific scan analyses, rather than a joint multi‑serotype model, represents a methodological limitation. Although this approach aligns with the structure of available surveillance data and preserves the interpretability of serotype‑specific clusters, it does not account for potential interactions between serotypes that may influence cluster detection under intense multi‑serotype transmission [40]. Future developments in multivariate spatial scan statistics may enable more rigorous joint inference

The use of the unweighted mean of annual municipal population projections as a fixed denominator introduces a modest and likely non-differential source of imprecision in incidence rate calculations, as this simplification does not account for year-to-year demographic change and may bias incidence estimates in municipalities with rapid population growth or decline. While SaTScan uses raw case counts as its primary input, which limits the impact on cluster detection, the reported incidence rates should be regarded as approximations, and year-specific denominators would be preferable in settings with marked demographic change.

Finally, the reliance on the municipality as the unit of spatial aggregation makes the analysis subject to the modifiable areal unit problem (MAUP), whereby the detection, size, and boundaries of clusters can depend on both the scale of aggregation and the specific zoning of the areal units [41,42]. Municipalities are administrative divisions that do not necessarily correspond to the ecological or epidemiological boundaries of transmission, and their substantial heterogeneity in geographic area and population size means that a single municipal centroid may inadequately represent transmission occurring within large or internally heterogeneous units. Aggregation at this resolution may therefore obscure finer-scale clustering at the locality or neighborhood level, where dengue transmission is known to operate [42], while a coarser scale, such as the state level, would mask the sub-national heterogeneity that this analysis was designed to capture. The municipal scale was selected as a deliberate compromise between epidemiological resolution and the spatial granularity of the publicly available surveillance data, but the detected clusters should be interpreted as features of this specific areal configuration rather than as scale-invariant transmission units.

These limitations indicate that the findings are best interpreted as a serotype-resolved description of where laboratory-confirmed dengue clustered under the prevailing surveillance and aggregation conditions, rather than as a complete or unbiased representation of national transmission, and they should inform, but not by themselves determine, public health prioritization.

## Conclusions

The 2020–2025 period represented one of the most complex dengue epidemiological periods in Mexico’s modern surveillance history, characterized by the reemergence of a long-absent serotype, the simultaneous circulation of all four DENV serotypes across a substantial share of the national territory, and the largest single dengue cluster ever documented in this dataset. Space-time scan analysis at municipal resolution, applied independently by serotype, provides the analytical resolution needed to distinguish the serotype-specific geographic and temporal fingerprints of transmission and to identify the municipalities where co-circulation conditions were most intensely and durably realized.

The 217 municipalities with documented four-serotype co-circulation represent a spatially defined priority tier for public health action. These localities concentrate the epidemiological conditions most consistently associated with severe dengue and should be the first targets for enhanced clinical surveillance, seroprevalence studies, and intensified vector control operations. The persistent transmission foci identified across multiple serotypes and epidemic cycles further argue for permanent entomological surveillance infrastructure in these municipalities, rather than reactive capacity deployed only during outbreak responses. Should dengue vaccination be incorporated into Mexico’s public health strategy in the future, the co-circulation maps generated by this approach could contribute to geographic prioritization, alongside other epidemiological and programmatic considerations.

These findings also show that serotype-resolved spatiotemporal analysis of existing surveillance data can generate actionable intelligence at no additional data collection cost. Replicating this framework across other endemic countries with comparable surveillance infrastructure would advance the regional capacity to anticipate severe dengue risk, monitor serotype dynamics, and allocate preventive resources where the probability of sequential heterotypic exposure is demonstrably highest.

## Supporting information

S1 File Supplementary_Dataset. (XLSX)
